# Supplementary material for: Near-Death Quality of Life in Cancer Patients on Home Parenteral Nutrition
Source: Nutrients. 2025 Jan 13;17(2):271. doi: 10.3390/nu17020271 (PMC11767650; doi:10.3390/nu17020271)
Supplement: Supplementary file 1 [file nutrients-17-00271-s001.zip › nutrients-3388400-supplementary.pdf]

Materials and Methods

2.1. Study design

Our inclusion criteria for accepting patients in the HPN program followed the guideline recommendations [1,2] and included: proven and prolonged failure to meet nutrition requirements by the oral or enteral route, with impending risk of death due to malnutrition; life expectancy >2 months; Karnofsky performance status (KPS) >50; control of pain; absence of severe organ dysfunctions; written informed consent confirming that the patient accepted this modality of nutrition support; approval by the physician responsible for HPN, the oncologist and the general practitioner; presence of environmental conditions compatible with HPN; availability of an in-home caregiver; and availability of a specifically trained nursing team dedicated to the patient home care, as provided by the Public Health Service. Primary intervention aims to prevent a potential risk of earlier death due to malnutrition rather than from cancer progression in aphagic or severely hypophagic, malnourished patients with cancer in who PN is the only viable feeding option.

Exclusion criteria for HPN were capability to meet the nutritional requirements by oral or enteral route; KPS <50; uncontrolled symptoms; severe organ dysfunctions (heart, respiratory, liver and renal); lack of an in-home caregiver and HPN refusal by the patient.

The inclusion and exclusion criteria for accepting patients in the HPN program used within this study represent the current standards of care within clinical practice in our region.

References

1. Staun, M.; Pironi, L.; Bozzetti, F.; Baxter, J.; Forbes, A.; Joly, F.; Jeppesen, P.; Moreno, J.; Hébuterne, X.; Pertkiewicz, M.; Mühlebach, S.; Shenkin, A.; Van Gossum, A. ESPEN Guidelines on Parenteral Nutrition: home parenteral nutrition (HPN) in adult patients. *Clin Nutr*. **2009**, 28(4), 467-79.
2. Bozzetti, F.; Arends, J.; Lundholm, K.; Micklewright, A.; Zurcher, G.; Muscaritoli, M.; ESPEN. ESPEN Guidelines on Parenteral Nutrition: Non-surgical oncology. *Clin Nutr*. **2009**, 28(4), 445-54.
